# Weak central coherence in neurodevelopmental disorders: a comparative study

**DOI:** 10.3389/fpsyg.2024.1348074

**Published:** 2024-06-12

**Authors:** Leyre Gambra, Sara Magallon, Nerea Crespo-Eguílaz

**Affiliations:** ^1^Faculty of Education and Psychology, University of Navarra, Pamplona, Spain; ^2^Pediatric Neurology Unit, Clinic University of Navarra, Pamplona, Spain; ^3^Facultad de Educación, Universidad Internacional de La Rioja, Logroño, Spain

**Keywords:** central coherence, non-verbal learning disorder, attention deficit and hyperactivity disorder, social communication disorder, autism

## Abstract

**Introduction:**

Central coherence is the normal tendency to process and give meaning to incoming information taking into account the context or global view of that information.

**Methods:**

We assessed the central coherence of 252 school children of normal intelligence between 6 and 11 years old. We compared the performance of two groups: (a) a control group (*n* = 194), and (b) a clinical group (*n* = 58) comprising children with NVLD+ADHD (*n* = 24), ADHD alone (*n* = 16), SCD (*n* = 8) and level-1ASD (*n* = 10) (Kluskall-Wallis H and Mann-Whitney U were calculated to make comparisons within groups and between pairs of groups). The effects of medication were studied (Student’s *t* test).

**Results:**

The NVLD+ADHD, SCD and ASD1 groups showed weak central coherence. The performance of the ADHD group was normal and differed significantly from the NVLD+ADHD group.

**Conclusion:**

Central coherence deficit was not exclusive to ASD1: it also characterizes NVLD and SCD.

## 1 Introduction

The world is perceived as being hierarchically organized and includes global perceptions comprising local details (D’Souza et al., 2016); a human being is able to process information at both global and local levels. This capacity, which is denominated central coherence, is implied in everyday activities such as categorization, inspection of details in our surroundings, perception of the structure of a scene and analysis of information (Nayar et al., 2017). Central coherence is, therefore, the normal tendency to process and give meaning, in a global manner, to incoming information in its context (Noens and van Berckelaer-Onnes, 2008). It is thanks to central coherence that when we receive a message, we prioritize comprehension of meaning and not just the literal form (Crespo-Eguílaz et al., 2012).

Attwood (Attwood, 2007) provides a metaphor that may be useful to understand the nature of weak central coherence: “imagine rolling up a sheet of paper to form a tube and with one eye closed bring it up to other open eye, as if it were a telescope, and look at the world through it: you see the details but do not perceive the context.” A person with difficulty in central coherence (that is, a preference for analytical processing rather than global processing) has specific difficulties in simultaneously processing information perceived and in giving coherent, integral meaning to that information. The cognitive style of such a person is, therefore, characterized by a tendency to process details (Navon, 1977; Schooler, 2002; Förster and Dannenberg, 2010; Crespo-Eguílaz and Narbona, 2011) This style of processing is slower and more demanding from the cognitive point of view (Nayar et al., 2017). An ability to carry out tasks requiring central coherence rapidly is of fundamental importance in learning and in social behavior (Crespo-Eguílaz et al., 2012). Therefore, weakness in central coherence entails difficulties in contextual comprehension of social situations and in adaption to these situations (López and Leekam, 2007). Difficulties in central coherence are also described by (Lamb and Robertson, 1989) as local bias and by (Vermeulen, 2015), in his studies on people with autism spectrum disorders (ASD), as context blindness. To date, dysfunction in central coherence has principally been studied in ASD (López and Leekam, 2007), although it has also been described in other disorders, such as, Down’s syndrome, Williams syndrome (D’Souza et al., 2016) non-verbal learning disorder (NVLD) (Gillberg, 2003, 2009; Crespo-Eguílaz and Narbona, 2011); and thus weak central coherence is apparently not specific to ASD.

### 1.1 Central coherence in autism spectrum disorder

It has been demonstrated that people with ASD tend to process information in a manner that is more focussed on details than on the overall meaning, that is, they have weak central coherence (Frith, 1992; Nydén et al., 2011). Theory concerning central coherence is supported by studies that confirm that, in tests of local preference, people with ASD perform significantly better than people of normotypical development (Shah and Frith, 1983; Jolliffe and Baron-Cohen, 1997; Plaisted et al., 1998; O’Riordan et al., 2001; Pellicano et al., 2006), which demonstrates that people with ASD tend to focus their attention more on parts of objects than on the objects themselves (Ornitz et al., 1977). Other research has shown that subjects with ASD perform poorly in tests of global preference (Mottron and Belleville, 1993; Rinehart et al., 2000), that is, in tests that include tasks that require detection of relatively small visual elements embedded in large fields (Caron et al., 2006), of visual searching (O’Riordan et al., 2001), of pattern discrimination (Plaisted et al., 1998; Bertone et al., 2005) or that involve design of blocks, impossible figures or embedded figures (Happé and Frith, 2006). Some studies find that in ASD subjects there is hyperfunction of brain areas generally involved in primary perception in contrast to perceptual integration, and researchers have proposed that this hyperfunction might be the explanation for the perceptual endophenotype in autism. The special abilities of the so-called autistic savant and the variability across the spectrum of ASD are possible manifestations of a tendency to use primary perceptual functions (Mottron et al., 2006). Various studies of people with “high-functioning autism” have led to conclusions compatible with this hypothesis (Bertone et al., 2003; Wang et al., 2007; Koldewyn et al., 2013; Syriopoulou Delli et al., 2016): participants with autism perform well at tasks involving perception of faces in stationary images (photos of faces) (Lahaie et al., 2006), at tasks requiring perception of movement (Bertone et al., 2004) and at the Wechsler scale Cubes test (Caron et al., 2006).

A modified version of the central coherence theory has been proposed in which it is hypothesised that in individuals with ASD the bias towards local processing can be overcome when doing tasks with explicit demands for global processing (Happé and Frith, 2006). According to this model, people with ASD do not necessarily have difficulties in perceiving the global form of things but rather have an over-specialized perceptual system that, depending on the requirements of a task, can interfere with higher-level cognition (Mottron and Burack, 2001; Caron et al., 2006).

Finally, despite otherwise contradictory findings (Happé, 1999), it has been established that the local-precedence style of information processing is not universally present across the whole autism spectrum (Happé, 1999).

### 1.2 Central coherence in non-verbal learning disorder

Children with non-verbal learning disorder (NVLD)−also denominated Deficits in Attention, Motor control and Perception (DAMP) and Procedural Learning Disorder (PLD) (Crespo-Eguílaz and Narbona, 2009)−show signs of weak central coherence. Such children can get lost in details rather than process information in an integrated and correct way (Doty, 2019). They find it difficult or are slow to arrive at a coherent comprehension of complex images or scenarios (Crespo-Eguílaz and Narbona, 2009; Magallón, 2011; Crespo-Eguílaz et al., 2012). They tend not to understand globally but rather in parts, which makes it difficult for them to carry out integration of concepts and abstraction and, therefore, to make correct adaptation of understanding to context (Díaz Lucero et al., 2011); they have difficulty perceiving globally, analyzing, organizing and summarizing information (Chow and Skuy, 1999; Molenaar-Klumper, 2002; Mammarella and Pazzaglia, 2010). In addition, it has been found that these children can perform poorly in certain visual perception tasks (for example, in perceiving the spatial location of objects), and have difficulties in recognizing what is detail, simultaneous processing, combining parts into a whole, and visual-spatial organization (Schoemaker et al., 2001; Drumond et al., 2005). Sometimes, children with NVLD make errors in spatial perception, for example, mistaking places in their surroundings or in the position of a person relative to themselves (Viñuela, 2007). Consequently, they find it difficult to cope with novel environments and to solve problems that have a visual-spatial component (González, 2017). They tend to get lost in open, unstructured situations in which conversations often overlap, there is more use of colloquial language, many gestures are used and body distances need to be managed (Foss, 2001). They evidence both deficits in the comprehension of extra-verbal information (facial expressions, gestures, mimicry, body postures, prosodic inflections and other visual aspects of their circumstances) and also difficulties in integrating and understanding such information (Humphries et al., 2004; Mammarella et al., 2009). They find it challenging to adapt to novel situations and tend to make generalisations based on specific verbal utterances, without taking into account the context in which a conversation is taking place (Worling et al., 1999).

The above-mentioned impediments have a major impact on people with NVLD when it comes to giving meaning to different contexts and interpreting discourse and affects the contextualisation of language. They find it difficult to understand figurative language, irony and jokes; they may interpret language literally and have problems adapting to novel situations of social interaction (Semrud-Clikeman and Hynd, 1990; Colomé Roura et al., 2009; Narbona et al., 2011). Consequently, people with NVLD are unable to communicate effectively in everyday situations (Colomé Roura et al., 2009) and experience difficulties in social relations.

### 1.3 Central coherence in attention deficit and hyperactivity disorder

Booth and Happé (Booth and Happé, 2010) compared how well participants with ADHD, with ASD and with normotypical development performed at completing sentences or phrases (for example, “Hunting with a knife and … fork”); they found that the participants with ADHD correctly performed at this task, while the ASD group performed significantly worse than the control group.

In a study by Crespo-Eguílaz et al. (2012), 200 school children−20 with NVLD, 60 with ADHD, 60 with both NVLD and ADHD, and 60 controls - were given a test involving a chimerical image and an incoherent visual scene. Of the children with ADHD, only 8% failed in rapid interpretation of the chimerical image, and only 7% performed poorly in comprehension of the visual scene. In a similar research (Magallón, 2011), found that only 13% of schoolchildren with ADHD performed badly in the chimerical image task. Similar findings are founded by different authors (Booth et al., 2003; Zhang and Adipat, 2005; Pina et al., 2013).

### 1.4 Central coherence in social communication disorder

Children with SCD tend to interpret language literally and not to detect irony, inference and/or metaphors (Bishop and Rosenbloom, 1987; Bishop and Adams, 1992; Leinonen and Letts, 1997; Bishop, 2000; Mulas Delgado et al., 2006; Velarde et al., 2017). They present problems in adapting their language to the needs of the listener or the situation, and they also lack flexibility when topics change during a dialogue (Rapin and Allen, 1983; McTear, 1985; Conti-Ramsden and Gunn, 1986; Bishop, 2000; Mendoza Lara and Muñoz López, 2005; González et al., 2015).

In the DSM-5 criteria for SCD (American Psychiatric Association [APA], 2013), defining characteristics of the disorder are deficiency in the use of communication for social purposes (such as, greeting people and sharing information in a manner appropriate to the social context or to the needs of the person listening) and difficulty understanding what is not said in an explicit manner and what is non-literal or ambiguous (for example, idioms and humor) (Monfort, 2001; Baixauli-Fortea et al., 2004; Perkins, 2010; Monfort Juárez Centro Entender Hablar I, 2013; Martínez Alonso et al., 2021).

Given this review of the literature, our objective is to confirm that schoolchildren with ASD and NVLD experience difficulties with central coherence. Additionally, we aim to test whether children with SCD exhibit weak central coherence, a hypothesis suggested by our clinical observations but not yet empirically verified. Furthermore, we will examine the performance of children with ADHD concerning this construct and determine whether the central coherence deficit observed in children with NVLD can be explained by their attention difficulties and/or hyperactivity.

## 2 Materials and methods

### 2.1 Participants

The study sample was of 252 participants comprising a control group of 194 normotypical schoolchildren and 58 children with clinical disorders recruited at the neuropediatrics unit of the Clínica Universidad de Navarra hospital. The disorders were non-verbal learning disorder in conjunction with attention deficit and hyperactivity disorder (NVLD + ADHD; *n* = 24); ADHD (*n* = 16); level 1 autism spectrum disorder (ASD1 = 10) and social communication disorder (SCD; *n* = 8). All children were at primary school, between 6 and 11 years old (Table 1), and had typical intelligence as evaluated by Raven’s Progressive Matrices Test (2001) (Table 1). The children were from families of middle to middle-high socio-economic and cultural level on the Hollingshead scale (Hollingshead, 1957). All the children were Caucasian in race.

**TABLE 1:** Sex distribution and IQ-related statistics for the study sample.

	Control group	Clinical sample			
		**NVLD+ADHD**	**ADHD**	**SCD**	**ASD1**
*n*	194	24	16	8	10
Male	76	22	14	4	8
Female	118	2	2	4	2
Ratio male: female	0.6:1	11:1	7:1	1:1	4:1
IQ: mean (SD)	–	100.4 (9.9)	104.8 (8.7)	101.7 (11.2)	100 (12.4)
IQ: min.-max.	–	82–129	83–114	83–119	87–119

The above data cannot be used to infer prevalence because the subjects in this study volunteered to participate: not all patients with these pathologies who attended our neuropediatrics unit chose to participate.

The proportions of boys and girls and IQ-related statistics are given in Table 1. IQ data was not available for the control group; exclusion criteria for the control group included low academic performance, learning difficulty or behavioural disorders as determined, at the time of the study, by teachers and other specialist education professionals. 45.8% of the participants with NVLD+ADHD and 62.5% of those with ADHD were receiving pharmacological treatment with methylphenidate to improve attention (Table 4). The questionnaire and methodology for this study was approved by the Human Research Ethics committee of the University of Navarra (Ethics approval number: 2017.004mod1).

Written informed consent was obtained from the parents (Tables 1, 2).

**TABLE 2:** Age distribution of the sample studied.

Age	Control group	Clinical sample	Clinical sample by pathology			
			**NVLD+ADHD**	**ADHD**	**SCD**	**ASD1**
6	36	5	2	0	1	2
7	39	9	2	4	1	2
8	40	8	4	2	1	1
9	16	11	4	6	0	1
10	38	7	3	2	1	1
11	25	18	9	2	4	3
Total	194	58	24	16	8	10

### 2.2 Tools and procedures

All children took the Central Coherence Test (CCT) (Gambra, 2020), which is an in-house development comprised of 36 items grouped in four dimensions, each of which has, in turn, different visual and verbal sub-tests or tasks (Table 3).

**TABLE 3:** Dimensions and subtests of the Central Coherence Test.

Dimensions	Subtests	*No* of items	Items
Inference		11	1–11
	Irony	3	1–3
	Literality	7	4–10
	Verbal story-telling	1	11
Verbal detail		6	12–17
	Nonsense questions	2	12–13
	Nonsense sentences	4	14–17
Simultaneity		9	18–26
	Chimerical images	4	18–21
	Inconsistent pictures	1	22
	Hidden numbers and colours	4	23–26
Context		10	27–36
	Inconsistent visual scenes	4	27–30
	The phone call	6	31–36
Total		36

Previous research has demonstrated the validity and reliability of the CCT (Gambra, 2020) by means of reliability analysis (Cronbach’s alpha and Spearman’s rank correlation coefficient) and establishing construct validity (exploratory and confirmatory factor analysis and other studies), and establishing convergent and discriminant validity.

The dimensions that make up the test, as well as the subtests of each dimension, are as follows:

(a) Inference: The tasks that make up this dimension assess the ability to give meaning to different sentences and texts according to the contextual situation in which they exist, as well as the ability to understand figurative language, irony and jokes.
  a. Irony: assesses the understanding of various ironic comments made in three different everyday situations.
  b. Literality: assesses the ability to understand the non-literal meaning of a series of sentences on the basis of the context in which each sentence occurs.
  c. Verbal story-telling: assesses ability to provide a coherent ending to incomplete stories.
(b) Verbal detail: this dimension evaluates ability to detect incoherent features in different situations presented verbally.
  a. Nonsense questions: assesses ability to detect coherence or incoherence in a series of nonsense questions.
  b. Nonsense sentences: assesses ability to detect inconsistency in sentences that are grammatically correct but that are inconsistent in terms of content. The nonsense sentences are mixed in with a series of sensible sentences, which serve as distractors.
(c) Simultaneity: this dimension assesses the ability and speed of the schoolchild in making sense of an inconsistent situation: a series of non-coherent images and texts.
  a. Chimerical images: these evaluate simultanagnosia. After seeing each chimeric image for two seconds, the child is asked whether he/she has identified both, one or none of the animals or objects that make up the chimera. The child is also asked to describe the inconsistency between the two components.
  b. Inconsistent pictures: these are used to evaluate ability to orally describe illustrated actions that are inherently inconsistent.
  c. Hidden numbers and colours: this test evaluates ability to perceive details (numbers and colours) within a whole (a story). The test is done both visually (the child sees and reads the story) and aurally (the child hears the story).
(d) Context: the subtests in this dimension evaluate ability to understand and freely describe, orally, a series of images and audio recordings in which various actions are represented.
  a. Inconsistent visual scenes: these evaluate ability to detect incongruities in various images.
  b. The phone call: this is a series of role-play situations to assess ability to adapt to different contexts (Table 3).

We used Kolmogorov-Smirnov and Shapiro-Wilk tests to determine whether variables had a normal distribution. In addition Levene’s test was used to assess whether variances for the different groups were equal. For each clinical group, values for each test variable were converted to typified scores (for subtests and dimensions of the CCT, and for the CCT as a whole). For each group the performance profile was prepared. The non-parametric Kluskall-Wallis H and Mann-Whitney U were calculated to make comparisons within groups and between pairs of groups. Also, in order to determine whether medication influences test performance, we used Student’s t test to compare the mean performance of participants under medication with the mean performance of those who were not.

## 3 Results

There were no significant differences in performance between medicated and non-medicated participants. This was the case for all dimensions of the CCT and for all tasks. The finding applies to the two clinical groups in which there were patients under pharmacological treatment: the NVLD+ADHD group and the ADHD group (Table 4).

**TABLE 4:** Influence of medication on performance in dimensions and tasks of the Central Coherence Test.

	NVLD + ADHD group	ADHD group
Not under medication (*n*)	13	6
Under medication (*n*)	11	10
Dimension/task of the Central Coherence Test:	Student’s *t* comparing mean performance of medicated and non-medicated children:	
Inference	0.352	0.310
Irony	0.247	0.480
Literality	0.811	0.628
Verbal story-telling	0.397	0.191
Verbal detail	0.756	0.051
Nonsense questions	0.894	0.610
Nonsense sentences	0.283	0.272
Simultaneity	0.519	0.595
Chimerical images	0.416	0.063
Inconsistent pictures	0.827	0.319
Hidden numbers and colours	0.436	0.484
Context	0.950	0.911
Inconsistent visual scenes	0.550	0.665
The phone call	0.168	0.262

There were no significant differences between clinical groups or the control group in performance in the Verbal detail dimension.

**FIGURE 1:**
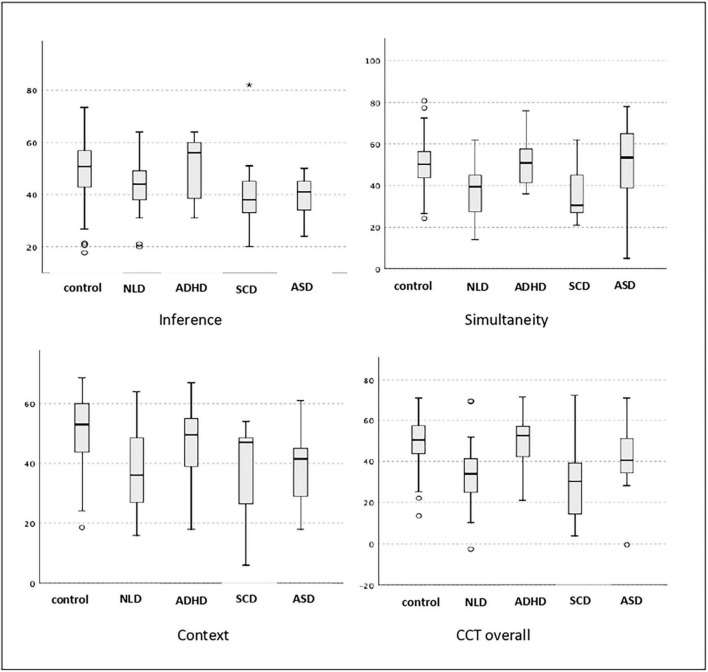
Comparison of performance levels of the clinical groups in the dimensions of the CCT and in the CCT as a whole.

With regard to the Simultaneity dimension, the performance levels of the NVLD+ADHD group and the SCD group were significantly poorer than that of the control group (Figure 1); the effect size is high (Table 5). Performance levels of the ADHD and ASD1 groups were typical (Figure 1). There were significant differences in this respect between the NVLD+ADHD group and the ADHD and ASD1 groups (Figure 1; Table 5).

**TABLE 5:** Comparisons of pairs of groups in performance in the dimensions of Inference, Simultaneity and Context and in overall CCT performance.

	χ	Sig.	Cohen’s D
**Inference**			
Control *vs.* NVLD+ADHD	46.52	**0.032**	6.79
Control *vs*. ADHD	−7.48	1	−0.56
Control *vs.* SCD	64.14	0.147	8.25
Control *vs.* ASD1	73.09	**0.020**	10.10
NVLD+ADHD *vs.* SCD	17.64	1	-
NVLD+ADHD *vs.* ASD1	26.58	1	-
NVLD+ADHD *vs.* ADHD	−54.00	0.217	-
SCD *vs.* ADHD	71.63	0.232	-
SCD *vs.* ASD1	8.95	1	-
ADHD *vs.* ASD1	80.58	0.061	-
**Simultaneity**			
Control *vs.* NVLD+ADHD	62.64	**0.001**	12
Control *vs.* ADHD	−5.32	1	−1.19
Control *vs.* SCD	74.8	**0.044**	14
Control *vs.* ASD1	−14.47	1	−0.30
NVLD+ADHD *vs.*SCD	12.17	1	-
NVLD+ADHD *vs.*ASD1	−77.11	**0.049**	-
NVLD+ADHD *vs.* ADHD	−67.96	0.**039**	-
SCD *vs.* ADHD	80.13	0.111	-
SCD *vs.* ASD1	−89.28	0.098	-
ADHD *vs.* ASD1	−9.15	1	-
**Context**			
Control *vs.* NVLD+ADHD	69.81	** < 0.001**	13.08
Control *vs.* ADHD	22.16	1	3.93
Control *vs.* SCD	59.13	0.245	12.75
Control *vs.* ASD1	66.31	**0.049**	11.34
NVLD+ADHD *vs.* SCD	−10.69	1	-
NVLD+ADHD *vs.* ASD1	−3.50	1	-
NVLD+ADHD *vs.* ADHD	−47.66	0.391	-
SCD *vs.* ADHD	36.97	1	-
SCD *vs.* ASD1	7.19	1	-
ADHD *vs.* ASD1	44.16	1	-
**Total CCT**			
Control *vs.* NVLD+ADHD	79.14	** < 0.001**	15.89
Control *vs.* ADHD	−3.10	1	−0.21
Control *vs.* SCD	84.80	**0.013**	19.36
Control *vs.* ASD1	44.24	0.622	9.39
NVLD+ADHD *vs.* SCD	5.67	1	-
NVLD+ADHD *vs.* ASD1	−34.90	1	-
NVLD+ADHD *vs.* ADHD	−82.24	**0.005**	-
SCD *vs.* ADHD	87.90	0.55	-
SCD *vs.* ASD1	−40.56	1	-
ADHD *vs.* ASD1	47.34	1	-

Significance values less than or equal to 0.05 are considered to be statistically significant. The Bonferroni correction for multiple-comparison testing has been applied to significance values. Effect sizes are interpreted as follows: *d* < 0.20: small; 0.20 < *d* < 0.80: average; *d* > 0.80: large. Bold values indicate statistically significant.

The performance of NVLD+ADHD and ASD1 groups in the Inference dimension was significantly lower than that of the control group, while the performance of the ADHD group was typical. The effect size of the differences was high (Table 5). The performance of the SCD group was also apparently lower, but this difference was not statistically significant (Figure 1).

In the Context dimension, the mean performance levels of the NVLD+ADHD group and the ASD1 group were significantly lower than that of the control group (Figure 1 and Table 5). Mean performance of subjects in ADHD and SCD groups was adequate. The effect size was high for all comparisons.

Finally, for the CCT as a whole, mean performance levels of the NVLD+ADHD group and of the SCD group were significantly lower (with large effect size) than that of the control group, while mean performance levels of the ADHD and ASD1 groups were average (Figure 1 and Table 5). Overall mean performance of the NVLD+ADHD group was significantly lower than that of the ADHD group (Table 5).

In total CCT score and in all dimensions except Verbal detail, the NVLD+ADHD group performed significantly worse than the control group. The performance of the ADHD group, however, was similar to that of the control group in all dimensions. Furthermore, the NVLD+ADHD and ADHD groups differed in overall CCT (Table 6). As the participants in both groups have attention deficit, we infer that the clear difficulties in central coherence in subjects with NVLD+ADHD is not to be explained by attention deficit but rather as being characteristic of NVLD.

**TABLE 6:** CCT performance profiles based on differences with respect to the control group.

	Relative to the control group		
	**NVLD+ADHD**	**ASD1**	**SCD**
Inference	average-low	average-low	average-low
Verbal detail	average	average-low	average-low
Simultaneity*	average-low	average	average-low
Context	average-low	average-low	average-low
CCT Total	low	average-low	average

A gray background indicates differences for which p < 0.001.

*In Simultaneity there was a statistically significant difference (*p* = 0.049) between NVLD+ADHD and ASD1 groups.

NVLD+ADHD and ASD1 groups had significantly lower mean performance than the control group in the Inference and Context dimensions (Table 6). The two groups differ in mean performance in Simultaneity: children with NVLD+ADHD performing worse than those with ASD1.

CCT performance profiles for NVLD+ADHD and SCD groups were similar. In both groups, mean performance in Simultaneity was significantly lower than that of the control group. However, the performance deficit of the NVLD+ADHD group was more serious than that of the SCD group because the symptomatology was more pronounced: the mean performance of the NVLD+ADHD group was significantly lower than that of the control group in most dimensions of the CCT (Table 6).

## 4 Discussion

The results of our study indicate that deficit in central coherence is not an effect exclusive to ASD. While various studies have found a reduction in global processing in ASD (Rinehart et al., 2000; Pellicano et al., 2005; Seernani et al., 2020) or that people with ASD perform significantly better in tests of local preference than people with typical development (Shah and Frith, 1983; Plaisted et al., 1998; O’Riordan et al., 2001; Pellicano et al., 2006; Gambra, 2020), there are also numerous studies that contradict such findings (Brian and Bryson, 1996) and that demonstrate that ability for global processing in people with ASD is intact (Bertone et al., 2003; Wang et al., 2004; Johnson et al., 2010).

Mottron and Belleville (Mottron and Belleville, 1993) demonstrate that people with autism process information, at both the local and the global level, as well as control subjects, but that in ASD the local interferes with the global when stimuli are incongruous. Subsequent studies confirm these findings (Jolliffe and Baron-Cohen, 1997; Rinehart et al., 2001). These somewhat contradictory findings have also been obtained in studies using more than one evaluation tool (Edgin and Pennington, 2005). Syriopoulou Delli et al. (2016) suggest that disparity in results might be best explained by considering that the style of information processing in autism is personal rather than a distinct characteristic of ASD.

In our research, the general performance of the ASD1 group was lower than that of the control group, but this difference was not always significant (depending on the dimension of the CCT); this is in agreement with the results of Syriopoulou Delli et al. (2016) who found that in a global preference task the scores of children with typical development were apparently higher than the scores of children with ASD, but the difference was not statistically significant. With regard to central coherence abilities, schoolchildren with ASD1 had average performance in simultaneity tasks (which were principally visual tasks) and average-low performance in tasks involving inference, verbal detail and understanding in context. Relative to the control group, differences in performance at tasks involving inference and context were significant. (The same differences were found in the NVLD+ADHD group, but children with ASD1 performed better than children with NVLD+ADHD in simultaneity tasks.)

In contrast to our findings, Loth (Loth, 2003) concluded that only about 35% of children with autism show weak central coherence in different tasks, while 48% have mixed styles of processing, with good performance in conceptual tasks. In our study, however, 90% of the schoolchildren in the ASD1 group had difficulty with one or more abilities related to central coherence. The difference in results can be explained in various ways. First, ASD is by nature heterogeneous and symptoms vary according to the level of ASD (Santangelo and Folstein, 1999; Tager-Flusberg and Joseph, 2003); in our study, all participants with ASD were level 1. Second, within ASD there can be subgroups defined by performance in terms of central coherence (Tager-Flusberg and Joseph, 2003). Third, as already discussed, the different tasks used by different studies make different demands on subjects and therefore give different measures of performance. Finally, Mottron et al. (Mottron et al., 2006) suggest that autism is often associated with improved perceptual processing, but this is not evident in all children with ASD.

Our study found that children with NVLD had difficulties with central coherence, confirming that this difficulty is a characteristic in the cognitive profile of NVLD. This finding is in line with different studies (Crespo-Eguílaz and Narbona, 2009; Magallón, 2011). Other authors (Chow and Skuy, 1999; Schoemaker et al., 2001; Molenaar-Klumper, 2002; Viñuela, 2007; Mammarella and Pazzaglia, 2010; Mammarella et al., 2019) do not refer explicitly to the construct of central coherence but nonetheless affirm that children with NVLD have difficulty with global perception and with analysing, organizing and synthesizing information.

General performance of the NVLD+ADHD group in central coherence tasks was lower than that of the control group (*p* < 0.001). However, performance was average for abilities related to verbal details. That is, for children with NVLD+ADHD, performance in tasks that involve verbal aspects of central coherence seems to stand out against the background of their overall performance. This concurs with the more general observation that the verbal abilities of children with NVLD+ADHD are better than their manipulative abilities. In our study, performance was average-low for abilities to understand in a simultaneous manner, to make inferences and to understand in a context (all of which were statistically significant differences relative to the control group). These results are coherent with those of Crespo-Eguílaz et al. (2012), who reported that rapid interpretation of a chimerical image - to make sense of which it was necessary to perceive and integrate both parts of the chimera - posed difficulty for 85% of schoolchildren with NVLD+ADHD but for only 5% of controls. Magallón (2011) found that children with NVLD+ADHD had difficulty in noticing incongruities in chimerical images (62.1% carried out this task badly in comparison to 8% of controls) and in visual scenes (about 60% performed poorly at this task). These results are along the same lines (Díaz Lucero et al., 2011), who evaluated the neuropsychological profile of a group of 22 children with DAMP and concluded that they did not understand globally but rather by parts, which made it difficult for them to integrate concepts, to carry out abstraction and, therefore, to adapt correctly to the context. Another research group, (Drumond et al., 2005), also found that children with NVLD had difficulty in tasks that involved construction of a whole from parts. Semrud-Clikeman et al. (2010) reported findings similar to ours for a group of children with NVLD (with difficulties in central coherence) relative to other groups of children with level-1 ASD and ADHD (without difficulties in central coherence).

In our study, the children with NVLD also had ADHD. However, as in the study of Makris et al. (2021), the difficulties in central coherence that we have discussed were not observed with a separate ADHD group. Therefore, these difficulties cannot be explained by attention deficit and appear to be characteristic of NVLD. In contrast to these results, (Cardillo, 2018) found that the central coherence profile of a group of children with ADHD was heterogeneous: the children had difficulty with visuoconstruction abilities when they had to battle with global configurations but performed visual-perception tasks correctly. In this study, the SCD group was characterized by average-low performance for all central coherence abilities studied. Thus, as a group, schoolchildren with a deficit in social communication skills have below-average performance, which indicates that their neuropsychological profile is also characterized by weak central coherence.

The study’s limitations include that all children in the NVLD sample have ADHD. It would have been preferable to identify another pure NVLD group, although this is challenging due to the frequent presence of comorbid disorders. Additionally, the sample size of the ASD group could be larger to draw definitive conclusions. Environmental or socio-economic factors not accounted for in the study could influence central coherence abilities. These factors might limit the generalizability of findings to different populations.

## 5 Conclusion

The current study, through the profiles of central coherence for the clinical groups described, is consistent with a deficit in central coherence is not exclusive to autism spectrum disorders. This study evidence that that children with other neurodevelopmental and learning disorders, such as, non-verbal learning disorder and social communication disorder, experience difficulties related with this cognitive function. In addition, it was found that schoolchildren with ADHD did not have difficulty with central coherence. Finally, we establish that the Central Coherence Test provides complementary information that is useful for differential diagnosis between neurodevelopmental disorders involving weak central coherence.

## Data availability statement

The raw data supporting the conclusions of this article will be made available by the authors, without undue reservation.

## Ethics statement

The studies involving humans were approved by the Ethics committee of the University of Navarra (Ethics approval number: 2017.004mod1). The studies were conducted in accordance with the local legislation and institutional requirements. Written informed consent for participation in this study was provided by the participants’ legal guardians/next of kin. Written informed consent was obtained from the minor(s)’ legal guardian/next of kin for the publication of any potentially identifiable images or data included in this article.

## Author contributions

LG: Writing–original draft, Conceptualization, Methodology, Writing–review and editing. SM: Formal analysis, Writing–original draft, Writing–review and editing. NC-E: Formal analysis, Writing–review and editing.
